# A brief review of bioluminescent systems (2019)

**DOI:** 10.1007/s00294-019-00951-5

**Published:** 2019-03-08

**Authors:** Aubin Fleiss, Karen S. Sarkisyan

**Affiliations:** 1 Synthetic Biology Group, MRC London Institute of Medical Sciences, London, UK; 2 Institute of Clinical Sciences, Faculty of Medicine, Imperial College London, London, UK; 3 Planta LLC, Bolshoi Boulevard, 42 Str 1, Office 335, Moscow, 121205 Russia; 4 Shemyakin-Ovchinnikov Institute of Bioorganic Chemistry of the Russian Academy of Sciences, Miklukho-Maklaya, 16/10, Moscow, 117997 Russia

**Keywords:** Bioluminescence, Luciferin, Luciferase, Synthetic biology, Imaging

## Abstract

Despite being widely used in reporter technologies, bioluminescent systems are largely understudied. Of at least forty different bioluminescent systems thought to exist in nature, molecular components of only seven light-emitting reactions are known, and the full biochemical pathway leading to light emission is only understood for two of them. Here, we provide a succinct overview of currently known bioluminescent systems highlighting available tools for research and discussing future applications.

## Introduction

Having evolved independently dozens of times, bioluminescence provides living organisms with a tangible advantage in certain ecological contexts. The ability to emit light in darkness has been observed in about 10,000 species from 800 genera, although this may well be an underestimation (Haddock et al. 2010). The exact benefit of light emission in various environments is far from being clear for a number of species, however, in most cases bioluminescence is thought to serve the purpose of visual communication to scare off predators, attract prey or in courtship behaviour (Ellis and Oakley 2016; Wainwright and Longo 2017; Verdes and Gruber 2017; Labella et al. 2017).

Evolution has stumbled upon and fixed numerous biochemical solutions for bioluminescence demonstrating that the ability to glow is accessible to living organisms in various points of genotype space, from bacteria to fungi and animals. Various *luciferins*, the small molecules prone to light emission upon oxidation, have been derived by evolution from unrelated biochemical pathways. Oxidation of these molecules is catalysed by non-homologous enzymes, *luciferases*, to create a palette of light-emitting reactions that are different in colour, catalysis rate, cellular localisation and dependence on ATP, NADH and other metabolites (Kaskova et al. 2016).

Although still largely understudied on a molecular level, hardly could such diversity of reactions with an easily measurable output escape becoming an essential part of modern reporter technologies. Luminescent reactions, where structures of both luciferin and luciferases have been discovered, are now utilised in vitro and in vivo in food testing (Shama and Malik 2013), environmental monitoring (Girotti et al. 2008), diagnostics (Frank and Krasitskaya 2014), drug screenings (Hasson et al. 2015; Kobayashi et al. 2010; Lampinen et al. 1995), and various kinds of biomedical research. Detailed reviews on chemistry and diversity of luciferins (Kaskova et al. 2016), luciferases (Kotlobay et al. 2019), and ecology of bioluminescence (Haddock et al. 2010; Widder 2010), as well as a comprehensive overview of all known bioluminescent systems (Shimomura and Yampolsky 2019), are available. In this article, we provide an intentionally succinct overview of light-emitting reactions where both luciferin and luciferase are known, highlighting their main features for practical applications.

**Fig. 1:**
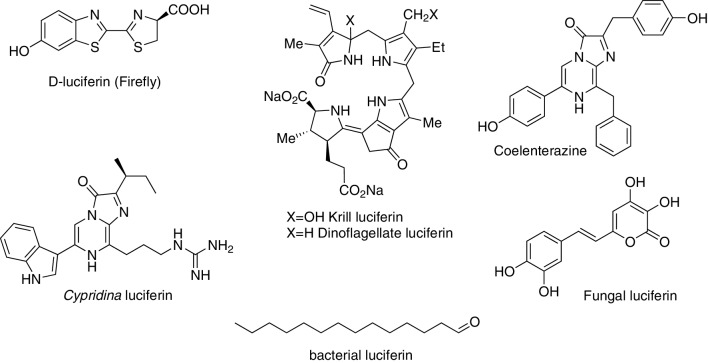
Structure of luciferins from bioluminescent systems with known luciferases

We will group bioluminescent systems by structures of their luciferins, as these compounds are the principal determinants of colour and properties of light-emitting reactions. Of at least forty bioluminescent systems thought to exist in nature (Haddock et al. 2010), structures of only nine luciferins are known, for seven of which at least a single luciferase gene has been discovered (Fig. 1).

## Main text

### Coelenterazine-dependent systems

The largest diversity of bioluminescent organisms is found in marine ecosystems. In the sea, it is often the same luciferin, *coelenterazine*, that serves as a substrate for numerous independently evolved luciferases in phylogenetically distant groups of organisms. Coelenterazine is a modified tripeptide produced from one phenylalanine and two tyrosine residues, however, the genes involved in its biosynthesis, as well as the exact biosynthetic route, are currently unknown. Most of marine organisms do not synthesise coelenterazine themselves, instead, they obtain it from food—the likely reason for a remarkable convergent evolution of bioluminescence in marine ecosystems. All coelenterazine-dependent systems from nature emit blue light, with emission maxima within the 450–500 nm range, and do not typically require any cofactors except for oxygen. In some cases, the colour of bioluminescence is altered by a fluorescent protein that interacts with the luciferase. Other characteristics such as molecular weight, pH-sensitivity, thermostability and catalysis rates of luciferases vary dramatically among coelenterazine-dependent systems. Below we highlight several practically important luciferases that utilize coelenterazine or its analogues.

- *Renilla luciferase*: a medium-sized (36 kDa) cytosolic protein from a coral that produces a steady luminescent signal. Early discovery (Lorenz et al. 1991), as well as the availability of engineered versions with increased brightness and red-shifted spectra (Markova and Vysotski 2015), made this system popular for biomedical applications, in particular, in bioimaging and drug-screening (Hamdan et al. 2005; Prinz et al. 2006).
- *Gaussia luciferase*: a small (20 kDa) secreted protein produced by a small crustacean from the group Copepoda, with high catalysis rate and exceptional thermostability. The activity of the protein depends on the formation of disulfide bonds making it unsuitable for certain heterologous systems. The signal of *Gaussia* luciferase scales linearly with the number of cells being assayed (Tannous et al. 2005; Chung et al. 2009) making this system useful for monitoring tumor progression and drug response.
- *Nanoluc luciferase*: an engineered variant of a luciferase from the shrimp *Oplophorus gracilirostris*. This small (19 kDa) protein utilises a cell-permeable coelenterazine analogue, lacks disulfide bonds, and produces a bright signal suitable for a broad range of applications. Fusions with fluorescent proteins result in bright engineered bioluminescent constructs with red-shifted spectra facilitating single-cell and whole-body bioluminescent imaging *in vivo* (Saito et al. 2012). One of the drawbacks of this system is the high cost of reagents.

### Cypridina luciferin-based system

Another modified tripeptide emitting blue light is cypridina luciferin, a metabolite found in the ostracod *Cypridina* and bioluminescent midshipman fish, *Porichthys*. Although the biosynthesis of this compound is also unknown, it was shown to be produced from tryptophan, isoleucine, and arginine (Oba et al. 2002). Among other applications, the *Cypridina* system has been widely used in bioimaging, in studies of circadian rhythms (Yamada et al. 2013; Watanabe et al. 2010; Noguchi et al. 2012), and in immunoassays (Miesenböck and Rothman 1997; Wu et al. 2007, 2009).

### D-luciferin-dependent systems

Another practically important group of bioluminescent reactions, the most thoroughly studied one, has evolved in several lineages of beetles, including fireflies, click beetles and railroad worms. These reactions utilise a stable and nontoxic compound (Deluca 1976; Tiffen et al. 2010) known as *d-luciferin* to emit yellow, orange and in some cases red light, and represent another intriguing example of an independent origin of the same bioluminescent system (Fallon et al. 2018). Most luciferases oxidizing D-luciferin are proteins of ~ 60 kDa that depend on ATP and Mg^2+^ to catalyze a reaction similar to that of aminoacyl-tRNA synthetases and acetyl-CoA ligases. Dependence of light emission on the concentration of ATP has allowed to use the system as an ATP indicator in a variety of applications ranging from studies of cancer metabolism (Patergnani et al. 2014) to monitoring bacterial contaminants in water (Frundzhyan and Ugarova 2007) and diagnostics based on levels of ATP in blood (Abraham et al. 2003). Practically important enzymes include:

- *Firefly luciferase*: very popular as a reporter molecule due to the early discovery, high quantum yield of bioluminescence, availability of thermostable mutant variants with enhanced spectral characteristics and ease of production in bacteria. Firefly luciferase has been extensively used in various *in vitro* and *in vivo* systems to detect pathogenic bacteria (Abe et al. 2012; Nakamura et al. 2011) and viruses (Zammatteo et al. 1995; Minekawa et al. 2013), to quantify protein–protein (Arai et al. 2002; Hattori et al. 2013; Radeck et al. 2017) and protein–ligand (Shekhawat and Ghosh 2011) interactions, and to assay metabolites involved in cell communication and cell signaling (Luker et al. 2008), among other applications.
- *Click beetle luciferases*: the second most popular group of D-luciferin-dependent luciferases is derived from the click beetle *Pyrophorus plagiophthalamus*. This species emits light using four types of luciferases with emission maxima ranging from green (540 nm) to orange-red (593 nm). The color variability, tolerance to a wide range of pH conditions and the availability of engineered variants make click beetle luciferases attractive for numerous applications (Hall et al. 2018). Engineered variants are available commercially, for example, Chroma-Luc luciferases offered by Promega.

### Tetrapyrrole-based luciferins

Dinoflagellates (protists) and Euphausiids (krill) utilise two very similar tetrapyrrole-based luciferins and form another large group of bioluminescent species. In dinoflagellates, bioluminescence is localised in special organelles, scintillons, and occurs as flashes of light triggered by electrical or mechanical stimulation. It is thought to serve as a defense mechanism making their attackers visible and attracting the attention of predators from higher trophic levels (Haddock et al. 2010). In Euphausiids, light emission occurs in complex organs with specialized lenses and ability to focus, yet the exact ecological role of this adaptation is unclear (Johnsen 2005). Luciferases from Dinoflagellates and Euphausiids are rarely used as tools in research due to the unavailability of synthetic luciferin.

### Bacterial bioluminescent system

All bioluminescent bacteria utilise the same unique mechanism for light emission, where photons are produced in a set of reactions requiring flavin mononucleotide (FMN), myristic aldehyde, oxygen and nicotinamide adenine dinucleotide (NADH). In the course of reactions, myristic aldehyde is oxidised and is thus known as luciferin, although the true light source in bacterial bioluminescence is the FMN derivative. Bacterial luciferases consist of two polypeptide chains that form a complex (75 kDa) and are encoded in the *lux* operon together with enzymes catalyzing luciferin biosynthesis. In most cases, bioluminescence is blue (~ 490 nm), however, both natural (Daubner et al. 1987) and engineered (Ke and Tu 2011) red-shifted versions of the bacterial system exist.

The full pathway of luciferin biosynthesis has been known since late 80 s making the *lux* operon the only genetically encodable bioluminescent system available in the last three decades (Meighen 1991). This allowed to use the system to engineer autonomously glowing organisms, including other bacteria (Belas et al. 1982; Francis et al. 2000), yeasts (Gupta et al. 2003), mammalian cell lines (Patterson et al. 2005), plants (Krichevsky et al. 2010) and others. However, no *brightly* bioluminescent multicellular organisms have been created, perhaps due to toxicity or inefficiency of the system in eukaryotes (Hollis et al. 2001). Consequently, in most applications of the system, living bacteria are utilised as a light source. Among the main applications of the system are the studies of antimicrobial drugs, bacterial infections and environmental monitoring (Björkman and Karl 2001). The brightest version developed to date is iLux (Gregor et al. 2018).

### Fungal bioluminescent system

In 2018, a biochemical pathway generating bioluminescence in fungi has been described in its entirety, providing the first genetically encodable pathway from eukaryotes (Kotlobay et al. 2018). Fungi utilise a simple α-pyrone *3-hydroxyhispidin* that is oxidised by an insoluble luciferase in a reaction that only requires oxygen and results in the emission of green light (~ 520 nm). The wild-type *Neonothopanus nambi* luciferase, nnLuz, is functional in a variety of heterologous systems, with the performance similar to that of the firefly luciferase (Kotlobay et al. 2018), (Sarkisyan, unpublished data). It has been shown that the expression of as few as three genes from the fungal bioluminescent system is sufficient to engineer other glowing eukaryotes.

## Final remarks

With no bioluminescent system suitable for any task and application, different light-emitting reactions occupy different niches in modern reporter technologies. In bioimaging, where applications of bioluminescence and fluorescence-based approaches overlap, the former is used in experiments that require high dynamic range, low background or deep-tissue imaging. Toxicity assays and studies of bacterial biology are typically based on the bacterial bioluminescent system while drug screenings often employ D-luciferin-dependent or coelenterazine-dependent systems. When selecting a luciferin–luciferase pair for a particular application, several criteria have to be taken into account including thermostability, pH optimum, protein size, cellular or extracellular location, aggregation properties, emission wavelength, intensity, rate of the reaction or dependence on ATP and other cofactors.

A recent discovery of a eukaryote-friendly genetically encodable pathway in fungi may stimulate the development of new bioluminescence-based technologies that would not require addition of the substrate. Expression of the fungal bioluminescent system may result in the generation of autonomously glowing animals and plants where light emission would be used to visualise development, report physiological changes, signal progression of pathological states, or simply serve esthetic purposes (Reeve et al. 2014; Kotlobay et al. 2018; Landau et al. 2009). We also envision that the discovery of the fungal pathway has the potential to bring autonomous bioluminescence beyond the use in reporter technologies towards engineering of light-based communications between cells, organisms or living and non-living systems.

The potential of bioluminescence-based tools in synthetic biology has only been marginally explored. Given the practical importance of light-emitting reactions, general appeal of glowing organisms and the scope of available methods in organic chemistry, metabolomics and genetics (Garrido-Cardenas and Manzano-Agugliaro 2017; Kaskova et al. 2016), the field of bioluminescence is surprisingly understudied. At the same time, with new insights into the photophysics, genetics and ecology of bioluminescence being made every year, engineering new light-emitting and light-communicating living systems is becoming more accessible than ever before.
